# Caracterização Histológica das Lesões da Valva Mitral de Pacientes com Cardiopatia Reumática

**DOI:** 10.36660/abc.20200154

**Published:** 2021-03-03

**Authors:** Nayana F. A. Gomes, Marcelo A. Pascoal-Xavier, Livia S. A. Passos, Thiago Mendonça Nunes Paula, João Marcelo de Souza Aguiar, Felipe Vieira Guarçoni, Maria Cecília Landim Nassif, Claudio Leo Gelape, Renato Braulio, Paulo Henrique N. Costa, Luiz Guilherme Passaglia, Raquel Braga Martins, Walderez O. Dutra, Maria Carmo P. Nunes

**Affiliations:** 1 Universidade Federal de Minas Gerais Belo HorizonteMG Brasil Universidade Federal de Minas Gerais, Belo Horizonte, MG - Brasil.

**Keywords:** Doenças Reumáticas, Calcificação, Fibrose, Inflamação, Febre Reumática, Miocardite, Histologia, Estenose Mitral, Ecocardiografia/métodos

## Abstract

**Fundamentos:**

Os mecanismos subjacentes pelos quais a doença cardíaca reumática (DCR) levam à disfunção valvar grave não são totalmente compreendidos.

**Objetivo:**

O presente estudo avaliou as alterações histopatológicas nas valvas mitrais (VM) buscando uma associação entre o padrão de disfunção valvar predominante e os achados histopatológicos.

**Métodos:**

Em 40 pacientes submetidos à troca da VM devido a DCR e em 20 controles submetidos a transplante cardíaco, foram analisados os aspectos histológicos da VM excisada. Dados clínicos e ecocardiográficos também foram coletados. As análises histológicas foram realizadas usando coloração com hematoxilina-eosina. Determinou-se inflamação, fibrose, neoangiogênese, calcificação e metaplasia adiposa. Valores de p<0,05 foram considerados estatisticamente significativos.

**Resultados:**

A idade média dos pacientes com DCR foi de 53±13 anos, sendo 36 (90%) do sexo feminino, enquanto a idade média dos controles foi de 50±12 anos, semelhante aos casos, sendo a maioria do sexo masculino (70%). O endocárdio valvar reumático apresentou espessura maior que os controles (1,3±0,5 mm versus 0,90±0,4 mm, p=0,003, respectivamente), e infiltrado inflamatório mais intenso no endocárdio (78% versus 36%; p=0,004), com predominância de células mononucleares. Ocorreu fibrose moderada a acentuada mais frequentemente em válvulas reumáticas do que em válvulas controle (100% vs. 29%; p<0,001). Ocorreu calcificação em 35% das valvas reumáticas, principalmente entre as valvas estenóticas, associada à área valvar mitral (p=0,003).

**Conclusões:**

Apesar do intenso grau de fibrose, o processo inflamatório permanece ativo na valva mitral reumática, mesmo em doença tardia com disfunção valvar. A calcificação predominou em valvas estenóticas e em pacientes com disfunção ventricular direita.

## Introdução

A doença cardíaca reumática (DCR) continua sendo um grande problema de saúde pública, principalmente em países de baixa e média renda, onde é a principal causa de morte cardiovascular em crianças e jovens.^1^^–^^3^ Estimase que 33 milhões de indivíduos vivam atualmente com DCR, sendo responsável por mais de um milhão de mortes prematuras anualmente.^1^ A DCR tem o maior índice de anos de vida perdidos ajustados por incapacidade (em inglês, *Disability Adjusted Life Years* — DALY) relacionado a doenças cardiovasculares em crianças em todo o mundo.^4^ Grande parte da morbidade e mortalidade da DCR pode ser prevenida, mas se não tratada, insuficiência cardíaca e óbito são inevitáveis.^5^^,^^6^ A maioria dos óbitos ocorre em jovens, que de outra forma estariam nos anos mais produtivos de suas vidas, indicando o impacto devastador dessa doença.^6^^,^^7^

A DCR é uma sequela pós-infecciosa da febre reumática aguda (FRA) resultante de uma resposta imune anormal a uma faringite estreptocócica que desencadeia dano valvar.^8^ Ao contrário do miocárdio e do pericárdio, o tecido valvar frequentemente sofre dano permanente após a cardite inicial ativa.^9^ No estágio final da DCR, a inflamação crônica contínua segue ocasionando a remodelação patológica da valva que perpetua o dano valvar com o tempo.^10^^,^^11^ A DCR afeta, de maneira mais usual e severa, a valva mitral (VM) que, com o tempo, torna-se disfuncional, contribuindo para o aumento do risco de óbito e outros desfechos adversos importantes.

Apesar do progresso observado nas pesquisas sobre a patogênese da DCR, permanece uma série de questões científicas importantes.^12^^,^^13^ Especificamente, os mecanismos subjacentes envolvidos no desenvolvimento da disfunção valvar grave não são completamente compreendidos.^14^ Um processo inflamatório ativo e a ativação endotelial são fundamentais para perpetuar a remodelação progressiva do folheto fibrótico e posterior disfunção valvar.^15^ Portanto, uma melhor compreensão desse processo patológico que leva ao desenvolvimento de disfunção valvar severa fornecerá *insights* sobre a patogênese da doença e poderá, em última instância, levar a estratégias terapêuticas mais eficazes para prevenir danos valvares irreversíveis. No presente estudo, supomos que o processo inflamatório persista mesmo em estágios mais avançados da doença, o que contribui para a lesão valvar progressiva na DCR. O objetivo deste estudo é avaliar as alterações histopatológicas das valvas mitrais em um estágio final da disfunção valvar, buscando uma associação entre o padrão de disfunção valvar predominante e os achados histopatológicos.

## Métodos

### População de Estudo

Foram coletadas 60 valvas mitrais de janeiro de 2015 a 2018, sendo que 40 eram de pacientes com DCR e 20 do grupo controle, que incluiu todos os pacientes submetidos a transplante cardíaco por insuficiência cardíaca grave, sem lesão valvar primária.

Foram elegíveis para o estudo pacientes encaminhados ao Hospital Universitário da Universidade Federal de Minas Gerais (HC-UFMG) com diagnóstico de valvopatia mitral reumática, apresentando estenose ou regurgitação, e com indicação de cirurgia de troca valvar mitral.

Os pacientes foram informados sobre o estudo e convidados a participar, voluntariamente, durante o seguimento, antes do procedimento cirúrgico. Ofereceu-se tratamento a todos os pacientes, independentemente de sua disposição em participar do estudo, e todos os que concordaram em participar assinaram o termo de consentimento livre e esclarecido. O presente estudo foi aprovado pelo Comitê de Ética da Universidade Federal de Minas Gerais.

Realizou-se consulta clínica, anamnese e exame físico para coleta de dados clínicos prévios à cirurgia. O ecocardiograma foi feito no Setor de Ecocardiografia do HCUFMG, para coleta de dados ecocardiográficos e de imagem.

O manejo dos pacientes e a indicação de troca valvar mitral seguiram as diretrizes recomendadas para valvopatia.^16^^,^^17^

As cirurgias foram realizadas no Centro Cirúrgico do HCUFMG e as válvulas encaminhadas ao Serviço de Patologia do HC-UFMG para exame histopatológico convencional, conforme rotina estabelecida pelo Serviço de Cirurgia Cardiovascular e Cardiovascular do HC-UFMG, bem como do Laboratório de Patologia Molecular (LPM) do Departamento de Anatomia Patológica e Medicina Legal da FM-UFMG, para a realização deste estudo.

### Avaliação Ecocardiográfica

Imagens de ecocardiografia bidimensional (2D) e Doppler foram realizadas usando um sistema comercialmente disponível (Philips ie33, Andover, MA ou GE Vivid-q Horten, Noruega) em todos os pacientes. Os ecocardiogramas padrão foram obtidos de acordo com as diretrizes da Sociedade Americana de Ecocardiografia.^18^ Foram medidos os índices convencionais para avaliação da gravidade da estenose mitral, incluindo área da valva mitral, gradientes de pressão da valva transmitral e pressão sistólica da artéria pulmonar, conforme recomendado.

### Análise Histológica

As valvas recebidas no LMP foram examinadas e encaminhadas para processamento histológico de acordo com os protocolos laboratoriais de rotina, incluindo fixação, inclusão em parafina, coloração com hematoxilina-eosina e análise microscópica. A análise semiquantitativa das amostras foi realizada por meio de uma escala.

Foram avaliados os parâmetros de intensidade da inflamação, identificação do tipo celular predominante e intensidade da fibrose, além da presença de neoangiogênese, calcificação e metaplasia adiposa.

A espessura endocárdica foi determinada em milímetros. A intensidade da inflamação e fibrose foi classificada de forma semiquantitativa como ausente, leve, moderada e grave por 2 observadores independentes com análise subsequente por um patologista experiente que fez a classificação final. A presença de neoangiogênese, calcificação e metaplasia adiposa foi definida como ausente ou presente.

### Análise estatística

Variáveis categóricas, expressas como números e porcentagens, foram comparadas usando o teste quiquadrado. A normalidade das variáveis contínuas foi testada pelo teste de Shapiro-Wilk, e os dados com distribuição normal foram expressos como média±desvio padrão (DP) e as diferenças entre as categorias de intensidade da inflamação, área valvar e espessura do folheto foram avaliadas pelo teste t de Student não pareado. A área média valvar e a espessura do folheto foram comparadas em 3 categorias de inflamação, usando o teste ANOVA de uma via e a análise post hoc com o teste de Tuckey. Valores de p<0,05 foram consideradas estatisticamente significativas. A análise estatística foi realizada com o programa Statistical Package for Social Sciences for Windows, versão 22.0 (SPSS Inc., Chicago, Illinois).

## Resultados

### Características dos pacientes

**Figura 1:**
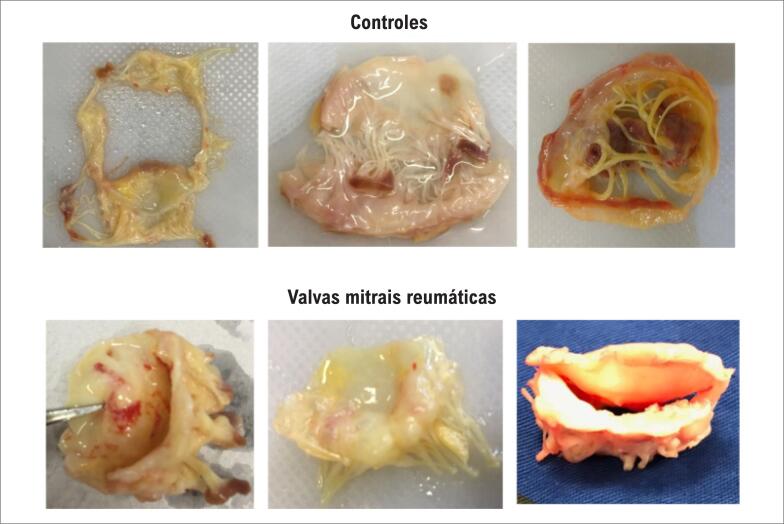
Aspectos morfológicos macroscópicos das valvas mitrais de controles e pacientes com cardiopatia reumática. Observe que as valvas reumáticas e os folhetos mitrais anterior e posterior se fundem nas comissuras. Cordoalhas encurtadas e fundidas.

A média de idade dos pacientes foi 53±13 anos e 36 (90%) eram do sexo feminino. As características da população estudada comparando pacientes com controles estão resumidas na Tabela 1. A maioria dos pacientes encontrava-se em classe funcional III e IV da NYHA (60%). A Figura 1 mostra fotos representativas das características anatômicas das valvas mitrais de controles e pacientes.

**Tabela 1:** Dados demográficos, clínicos e ecocardiográficos dos pacientes submetidos à troca valvar mitral devido a cardiopatia reumática comparando-se com controles submetidos a transplante cardíaco

Variáveis*	Valvas mitrais reumáticas (n=40)	Controles (n=20)	Valor de p
Idade (anos)	53±13,2	50±11,6	0,334
Sexo feminino	36 (90)	6 (30)	<0,001
Classe funcional da NYHA, III/IV	24 (60)	16 (80)	0,003
Insuficiência cardíaca direita	12 (30)	12 (60)	0,030
Valvoplastia mitral prévia	22 (55)	…	…
Histórico de febre reumática aguda	27 (68)	…	…
Uso de profilaxia antibiótica	13 (33)	…	…
Fibrilação atrial	24 (60)	5 (25)	0,003
Acidente vascular cerebral prévio	12 (30)	4 (20)	0,102
Hospitalização prévia por insuficiência cardíaca	17 (43)	20 (100)	<0,001
**Parâmetros ecocardiográficos**			
Diâmetro diastólico final ventricular esquerdo (mm)	51,7±10,9	66,3±7,6	<0,001
Diâmetro sistólico final ventricular esquerdo (mm)	35,8±8,6	57,1±8,2	<0,001
Fração de ejeção do ventrículo esquerdo (%)	60,6±10,5	26,5±7,5	<0,001
Área da valva mitral (cm^2^)	1,18±0,37	…	…
Diâmetro atrial esquerdo (mm)	55,1±10,5	48,9±6,4	0,006
Pressão sistólica da artéria pulmonar (mmHg)	46,5±18,3	39,7±16,8	0,292
Disfunção ventricular direita	16 (40)	9 (45)	0,785

* Os dados são expressos como média±DP ou número (porcentagem) de pacientes.

Os medicamentos pré-operatórios mais usados foram diuréticos (60%) e betabloqueadores (55%). Pacientes com fibrilação atrial e/ou AVC prévio faziam uso de anticoagulantes. Treze pacientes (33%) faziam uso de penicilina benzatina para prevenção secundária da febre reumática. A média de idade desses pacientes foi 47,9±11,3. Nenhum dos pacientes incluídos no estudo tinha evidência clínica de febre reumática aguda ativa.

Em relação ao padrão de envolvimento valvar, 18 pacientes (45%) apresentavam estenose mitral pura, 14 pacientes (35%) apresentavam valvopatia mitral mista e 8 pacientes (20%) apresentavam regurgitação mitral, predominantemente. Detectou-se lesão aórtica no ecocardiograma de 15 pacientes (38%). Havia sido realizada valvoplastia em 22 pacientes (55%), incluindo intervenção percutânea ou cirúrgica. A indicação cirúrgica para complicações da valvoplastia percutânea ocorreu em 6 pacientes (15%), 4 dos quais devido a insuficiência mitral grave relacionada a comprometimento de folheto ou aparelho subvalvar. Entre aqueles que desenvolveram insuficiência mitral grave, 2 pacientes apresentaram ruptura do folheto anterior e foram submetidos à cirurgia de emergência para troca valvar. Dois pacientes apresentaram laceração dos *scallops* P3 e A3 em contiguidade com comissura posteromedial e folheto posterior no local do *scallop* central (P3), respectivamente, e foram submetidos a cirurgia eletiva para troca valvar. Os outros 2 pacientes apresentaram piora da regurgitação mitral após o procedimento com abertura valvar subótima.

O grupo controle foi constituído por 20 pacientes submetidos a transplante cardíaco, com média de idade de 50±12 anos, semelhante aos casos de DCR, sendo a maioria do sexo masculino (70%). As causas da insuficiência cardíaca foram cardiomiopatia dilatada chagásica (9 pacientes), cardiomiopatia pós-miocardite (3), cardiomiopatia isquêmica (3), cardiomiopatia dilatada idiopática (4) e retransplante por rejeição autoimune ao enxerto (1). Encontrou-se regurgitação mitral secundária moderada a grave em 7 pacientes (35%). Os outros dados demográficos são apresentados na Tabela 1.

### Análise histopatológica de valvas mitrais reumáticas

**Figura 2:**
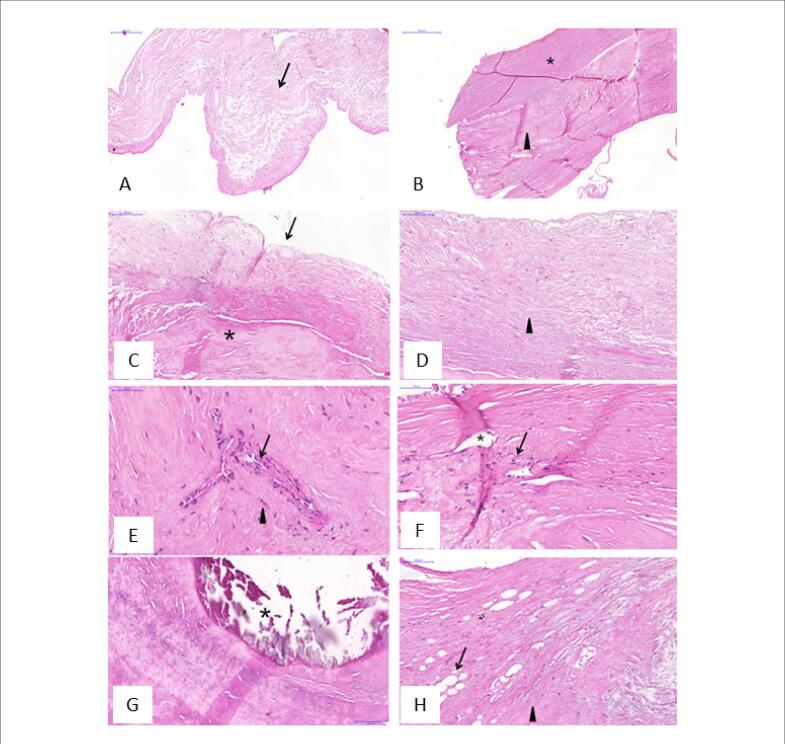
Imagem histológica representativa da valva mitral de controles e pacientes com doença cardíaca reumática corados com hematoxilina-eosina. A) Visão histológica de uma válvula mitral de controle, mostrando fibrose leve (seta). B) Valva mitral reumática com fibrose endocárdica (asterisco) e intersticial (ponta de seta) importante. Os painéis C-I mostram aspectos específicos das valvas mitrais de pacientes com doença cardíaca reumática. C) Presença de endocárdio (seta) e interstício valvar (asterisco). No endocárdio, há fibrose leve. D) Fibrose endocárdica moderada (ponta de seta). Coloração: hematoxilina-eosina. E) Neovascularização (seta) com alguns elementos inflamatórios (ponta de seta). F) Os focos inflamatórios (seta) são detectáveis dentro dos focos de neovascularização (asterisco). G) Calcificação nodular (asterisco). H) Metaplasia adiposa (seta) com áreas de fibrose (ponta de seta).

O endocárdio com valvas mitrais reumáticas era mais espesso (1,3±0,5 vs. 0,9±0,4 mm) com maior intensidade de fibrose e infiltrado inflamatório em comparação com os controles (Figuras 2A e B). Os achados histológicos das valvas mitrais reumáticas em comparação aos controles encontramse na Tabela 2. No geral, as valvas reumáticas apresentavam inflamação de intensidade leve, distribuição focal e predomínio de células mononucleares e fibrose de intensidade moderada a grave (Figuras 2C e D). Entre os pacientes em uso de penicilina benzatina, 9 (69%) apresentaram inflamação leve. A neoangiogênese foi mais frequente nas valvas mitrais reumáticas do que nos controles (Figuras 2E e F).

**Tabela 2:** Dados histológicos de valvas mitrais reumáticas e controles

Variável*		Valvas mitrais reumáticas (n=40)	Controles (n=20)	Valor de p
Endocárdio				
Espessura (mm)		1,3±0,5	0,9±0,4	0,003
Intensidade da inflamação	Ausente ou leve	9 (22)	13 (64)	0,004
	Moderada ou grave	31 (78)	7 (36)	
Padrão de inflamação	Focal	31 (78)	7 (36)	0,004
Intensidade da fibrose	Ausente ou leve	17 (43)	19 (95)	0,001
	Moderada ou grave	23 (57)	1 (5)	
Padrão da fibrose	Regular	23 (57)	0	< 0,001
	Irregular	17 (43)	7 (35)	
Neoangiogênese		11 (28)	0	0,030
**Interstício**				
Intensidade da inflamação	Ausente ou leve	33 (82)	14 (72)	0,321
	Moderada ou grave	7 (18)	6 (28)	
Padrão de inflamação	Focal	37 (92)	7 (36)	<0,001
Intensidade da fibrose	Ausente ou leve	0	14 (71)	<0,001
	Moderada ou grave	40 (100)	6 (29)	
Padrão da fibrose	Regular	4 (10)	0	0,200
	Irregular	36 (90)	20 (100)	
Neoangiogênese		24 (60)	7 (35)	0,130
Calcificação		14 (35)	0	0,010
Metaplasia adiposa		4 (11)	4 (21)	0,320

* Os dados são expressos média±DP ou número (porcentagem) de pacientes.

Nenhuma das valvas do grupo controle apresentou calcificação, enquanto 35% das valvas reumáticas apresentaram calcificação (Figura 2G). Apenas uma pequena porção das valvas em ambos os grupos apresentava metaplasia adiposa (Figura 2H).

De acordo com o tipo de lesão valvar mitral reumática, a calcificação foi mais frequente na estenose mitral pura em comparação com a regurgitação mitral ou lesões mistas. Os dados histológicos de acordo com a lesão valvar mitral predominante que necessitou de troca valvar estão apresentados na Tabela 3.

**Tabela 3:** Achados histológicos de acordo com a lesão valvar mitral predominante

Variável*		Estenose pura (n=18)	Regurgitação e lesões combinadas (n=22)	Valor de p
Inflamação	Ausente ou leve	15 (83)	18 (82)	0,900
	Moderada ou grave	3 (17)	4 (18)	
Neoangiogênese		11 (61)	13 (59)	0,897
Calcificação		10 (56)	4 (18)	0,014
Metaplasia adiposa		1 (6)	4 (18)	0,230

* Os dados são expressos como número (porcentagem) de pacientes.

**Figura 3:**
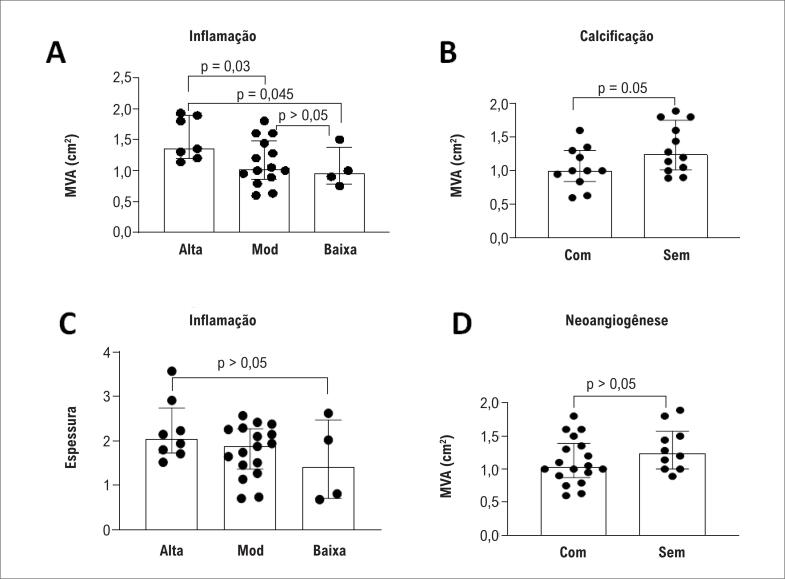
Associações entre achados histológicos e área da valva mitral e espessura do folheto. A) A valva mitral com grau alto e moderado de inflamação apresenta maior área valvar em comparação com válvulas com baixo grau de inflamação. B) A calcificação se mostrou associada à área valvar mitral, indicando a estenose mitral como lesão predominante nas valvas mais calcificadas. C) Associação entre a espessura do folheto e a intensidade da inflamação. D) Associação entre neoangiogênese e área valvar mitral.

Além disso, estratificamos os pacientes em 3 grupos de acordo com a intensidade da inflamação em baixa, moderada e alta para correlacionar com a área da válvula mitral. Observamos que os pacientes com maior grau de inflamação e sem calcificação apresentaram maior área valvar mitral (Figuras 3 A e B, respectivamente). Não houve diferenças em relação à espessura do folheto e intensidade da inflamação (Figura 3C), nem neoangiogênese e área valvar mitral (Figura 3D).

### Parâmetros ecocardiográficos associados a achados histológicos

Posteriormente, foram comparados os parâmetros ecocardiográficos e os achados histológicos. Observamos associação entre a função sistólica ventricular esquerda, avaliada pela fração de ejeção, e a intensidade da inflamação. Pacientes com disfunção ventricular esquerda, definida pela fração de ejeção menor que 50%, apresentaram predomínio de inflamação moderada a acentuada em relação aos pacientes com função sistólica ventricular preservada (50% e 12% respectivamente, p=0,023). Embora a calcificação valvar não estivesse associada à disfunção ventricular esquerda, pacientes com disfunção ventricular direita apresentaram calcificação maior em comparação com pacientes com função ventricular direita normal (56% e 21% respectivamente, p=0,021), o que pode estar relacionado ao tipo de lesão associado à calcificação.

## Discussão

A inflamação e a fibrose do tecido do folheto desempenham um papel central na indução de dano valvar progressivo na DCR.^13^ O presente estudo avaliou as valvas mitrais reumáticas no estágio final da DCR quando a disfunção valvar com risco de vida exigia cirurgia para troca valvar. Os achados histológicos das valvas reumáticas foram comparados com as valvas mitrais excisadas de pacientes submetidos a transplante cardíaco, sem doença valvar primária.

A análise dos dados histológicos mostrou fibrose acentuada entre todas as valvas reumáticas, mostrando que a fibrose contínua perpetua ao longo da progressão da DCR. O estado fibrótico da valva mitral precede a disfunção miocárdica e a doença cardíaca manifesta, tornando importante o desenvolvimento de estratégias diagnósticas e terapêuticas para reduzir a remodelação estrutural das valvas cardíacas.^19^

Notavelmente, embora os pacientes estejam no estágio mais avançado da doença, as valvas continuam apresentando processos inflamatórios ativos, predominantemente compostos por células mononucleares. A intensidade da inflamação mostrou-se associada à área da valva mitral, mas não à espessura do folheto. Encontrou-se baixa intensidade inflamatória em menor área valvar, provavelmente devido à intensidade da fibrose que tem associação inversa com o grau de inflamação.

Pacientes com disfunção ventricular esquerda apresentam maior grau de inflamação valvar, o que pode indicar miocardite adjacente. No entanto, 4 dos pacientes com disfunção ventricular esquerda tinham a insuficiência mitral como a lesão predominante que pode ser a causa da disfunção ventricular esquerda, o que é uma indicação para intervenção valvar.^16^^,^^17^

Estudos indicam que o processo autoimune envolvido na DCR começa quando os anticorpos reativos se ligam ao endotélio valvar, levando à inflamação e infiltração celular. Uma vez ativado, o endotélio valvar aumenta a expressão das moléculas de adesão, o que facilita a ligação e a infiltração das células T.^20^ Após o insulto valvar inicial, o processo desencadeia uma cascata que leva ao reconhecimento de epítopos adicionais, levando ao dano progressivo da valva.^21^ Evidências de que a apresentação contínua de autoantígenos no local da lesão contribui para uma amplificação da resposta imune são reforçadas pela redução significativa dos níveis de autoanticorpos após a remoção cirúrgica dos folhetos afetados.^22^

Após a ativação do endotélio valvar com a adesão das células T ativadas, inicia-se o ciclo de cicatrização, neovascularização e infiltração linfocitária.^23^ O tecido valvar avascular é normalmente protegido pelo endotélio até que um fator desencadeante, que podem ser anticorpos e/ou citocinas inflamatórias, rompe a barreira endotelial, permitindo o início do ciclo de infiltração e cicatrização celular.^24^ Uma vez iniciado, o processo de cicatrização torna-se mais intenso no interstício valvar devido à ativação e proliferação dos miofibroblastos responsáveis pela fibrose valvar.^25^ Já foi demonstrado que, diante de algumas patologias como a DCR, as células intersticiais podem se transformar em um fenótipo de miofibroblastos ativado, expressando proteínas inflamatórias e citocinas, capazes de remodelar rapidamente o meio extracelular.^26^ A redução significativa nos níveis plasmáticos de biomarcadores do metabolismo do colágeno após a troca da válvula mitral sugere fortemente a contribuição do aparelho da valva mitral para a perpetuação do processo fibrótico na DRC.^19^

Detectou-se calcificação em 35% das valvas reumáticas, predominantemente em estenose mitral pura e correlacionada com área valvar. A calcificação também se mostrou mais frequente em pacientes com disfunção ventricular direita. A identificação da calcificação reforça a cronicidade do processo, e sua ocorrência pode estar relacionada aos mecanismos subjacentes ao estágio final do comprometimento reumático.^27^ O predomínio de calcificações observadas em valvas reumáticas com estenose pura e em pacientes com acometimento do ventrículo direito confirma que essa lesão predomina nos estágios tardios da doença.^3^ Rajamannan et al.,^28^ demonstraram que a calcificação ocorre em áreas de neoangiogênese, estimulada por um processo inflamatório ativo, formado principalmente por macrófagos e miofibroblastos.^28^ Banerjee et al. encontraram maior grau de fibrose e neovascularização com infiltração leve perivascular focal predominantemente de linfócitos e células plasmáticas.^19^

### Limitações do estudo

O pequeno número de pacientes incluídos no estudo constitui uma limitação. Porém, considerando que a maioria dos pacientes reumáticos com indicação de intervenção valvar são submetidos à valvoplastia percutânea, o número incluído representa a totalidade das amostras disponíveis no período do estudo.

A alta prevalência de fibrilação atrial, histórico de acidente vascular cerebral, intervenção valvar prévia, dispneia limitante (NYHA III e IV) e hipertensão pulmonar mostram a gravidade dos pacientes com doença avançada. Como incluímos apenas pacientes com indicação de troca valvar, nossa população é, portanto, representativa de um espectro de estágios mais graves da doença.

A troca de valva mitral é indicada para pacientes sintomáticos com doença avançada e deformidade acentuada da anatomia valvar, onde o reparo da valva mitral é improvável.^29^ Portanto, a amostra coletada para o nosso estudo representa o processo reumático avançado, limitando a avaliação do processo em sua fase inicial.

As valvas do grupo controle não são saudáveis, normais, pois foram retiradas de pacientes submetidos a transplante cardíaco por disfunção ventricular esquerda grave e de diferentes etiologias. A cardiopatia chagásica, etiologia predominante das valvas coletadas em corações transplantados, é uma doença inflamatória, associada à fibrose. Embora a doença de Chagas afete principalmente o miocárdio ventricular, a alteração histológica da valva pode estar associada à insuficiência cardíaca.^30^ Além disso, 35% dos pacientes apresentavam insuficiência valvar mitral secundária moderada a grave, o que pode explicar os achados histológicos alterados. As diferenças na análise histológica podem ser ainda maiores se comparadas às valvas de tecido saudável.

### Implicações clínicas

O processo patológico envolvido na DCR é complexo e ainda não totalmente compreendido. A identificação de um processo inflamatório crônico ativo, embora de intensidade leve, provavelmente responsável pela manutenção da resposta imune e progressão da lesão valvar, fornece subsídios para pesquisas adicionais, no intuito de definir estratégias que possam interromper a progressão do dano valvar e suas consequências.

## Conclusões

Nossos achados demonstram que, apesar do intenso grau de fibrose, o processo inflamatório permanece ativo nas valvas mitrais reumáticas, mesmo no processo tardio da doença com disfunção valvar. Essa inflamação mostrou-se associada à área valvar mitral e à função ventricular esquerda. A calcificação valvar foi mais frequente na estenose mitral e nos pacientes com disfunção ventricular direita, indicando comprometimento reumático grave e tardio. É necessário entender melhor o que mantém a inflamação e como sua persistência pode predispor a complicações clínicas, bem como os mecanismos que influenciam a mortalidade por DCR.
