# Supplementary material for: Caracterização Histológica das Lesões da Valva Mitral de Pacientes com Cardiopatia Reumática
Source: Arq Bras Cardiol. 2021 Mar 3;116(3):404–12. [Article in Portuguese] doi: 10.36660/abc.20200154 (PMC8159546; doi:10.36660/abc.20200154)
Supplement: Translation

# Translation: Histopathological Characterization of Mitral Valvular Lesions from Patients with Rheumatic Heart Disease

**Authors:** Nayana F. A. Gomes, Marcelo A. Pascoal-Xavier, Livia S. A. Passos, Thiago Mendonça Nunes Paula, João Marcelo de Souza Aguiar, Felipe Vieira Guarçoni, Maria Cecília Landim Nassif, Claudio Leo Gelape, Renato Braulio, Paulo Henrique N. Costa, Luiz Guilherme Passaglia, Raquel Braga Martins, Walderez O. Dutra, Maria Carmo P. Nunes

**Affiliations:** 1 Belo HorizonteMG Brazil Universidade Federal de Minas Gerais, Belo Horizonte, MG - Brazil.

**Keywords:** Rheumatic Disease, Calcification, Fibrosis, Inflammation, Rheumatic Fever, Myocarditis, Histology, Mitral Valve Stenosis, Echocardiography/methods

## Abstract

**Background:**

The underlying mechanisms by which rheumatic heart disease (RHD) lead to severe valve dysfunction are not completely understood.

**Objective:**

The present study evaluated the histopathological changes in mitral valves (MV) seeking an association between the pattern of predominant valvular dysfunction and histopathological findings.

**Methods:**

In 40 patients who underwent MV replacement due to RHD, and in 20 controls that underwent heart transplant, histological aspects of the excised MV were analyzed. Clinical and echocardiographic data were also collected. Histological analyses were performed using hematoxylin-eosin staining. Inflammation, fibrosis, neoangiogenesis, calcification and adipose metaplasia were determined. A p value<0.05 was considered to be statistically significant.

**Results:**

The mean age of RHD patients was 53±13 years, 36 (90%) were female, whereas the mean age of controls was 50±12 years, similar to the cases, with the majority of males (70%). The rheumatic valve endocardium presented greater thickness than the controls (1.3±0.5 mm versus 0.90±0.4 mm, p=0.003, respectively), and a more intense inflammatory infiltrate in the endocardium (78% versus 36%; p=0.004), with predominance of mononuclear cells. Moderate to marked fibrosis occurred more frequently in rheumatic valves than in control valves (100% vs. 29%; p<0.001). Calcification occurred in 35% of rheumatic valves, especially among stenotic valves, which was associated with the mitral valve area (p=0.003).

**Conclusions:**

Despite intense degree of fibrosis, the inflammatory process remains active in the rheumatic mitral valve, even at late disease with valve dysfunction. Calcification predominated in stenotic valves and in patients with right ventricular dysfunction.

## Introduction

Rheumatic heart disease (RHD) remains a major public health concern, especially in low- and middle-income countries, where it is the leading cause of cardiovascular death in children and young adults.^1^^–^^3^ It is estimated that 33 million individuals currently live with RHD, accounting for over a million premature deaths annually.^1^ RHD has the highest cardiovascular disease-related loss of disability-adjusted-life-years (DALY) in children worldwide.^4^ Much of the morbidity and mortality of RHD can be prevented, but if left untreated, subsequent heart failure and death are inevitable.^5^^,^^6^ Most deaths occur in young adults, who would otherwise be at the most productive years of their lives, indicating the devastating impact of this condition.^6^^,^^7^

RHD is a harmful post-infectious sequel of acute rheumatic fever (ARF) resulting from an abnormal immune response to a streptococcal pharyngitis that triggers valvular damage.^8^ Unlike myocardium and pericardium, the valvular tissue often sustains permanent damage after active initial carditis.^9^ In the late stage of RHD, ongoing chronic inflammation continues leading to pathological valve remodeling that perpetuates valve damage over time.^10^^,^^11^ RHD most commonly and severely affects the mitral valve (MV) which, over time becomes dysfunctional, contributing to an increased risk of death and other major adverse outcomes.

Despite the observed progress in research on RHD pathogenesis, a number of key scientific questions remain.^12^^,^^13^ Specifically, the underlying mechanisms involved in the development of severe valve dysfunction are not completely understood.^14^ An active inflammatory process and endothelial activation are fundamental to perpetuate the progressive fibrotic leaflet remodeling and further valve dysfunction.^15^ Therefore, an improved understanding of this pathological process that leads to the development of severe valve dysfunction will provide insights into disease pathogenesis, and can ultimately lead to more effective therapeutic strategies to prevent irreversible valvular damage. In the present study, we hypothesized that the inflammatory process persists even in more advanced stages of the disease process, which contributes to progressive valve injury in RHD. The aim of this study is to evaluate the histopathological changes in mitral valves at an end stage of valve dysfunction, seeking an association between the pattern of predominant valvular dysfunction and histopathological findings.

## Methods

### Study population

A total of 60 mitral valves were collected from January 2015 to 2018, 40 were from RHD patients, and 20 were in the control group, which included all patients who underwent heart transplant due to severe heart failure, without primary valve lesion.

Patients referred to the University Hospital of Universidade Federal de Minas Gerais (HC-UFMG) diagnosed with rheumatic mitral valve disease, presenting stenosis or regurgitation, and with indication to mitral valve replacement surgery were eligible for the study.

The patients were informed about the study and invited to participate, voluntarily, during their follow-up before the surgical procedure. Treatment was offered to all patients, regardless of their willingness to participate in the study, and all those who agreed to participate signed an informed consent form. This study was approved by the Ethics Committee of Universidade Federal de Minas Gerais.

Clinical consultation, anamnesis and physical examination were performed to collect the clinical data prior to surgery, and the echocardiogram was performed at HCUFMG Echocardiography Sector, for the collection of echocardiographic and imaging data.

Patient management and indication for mitral valve replacement was according to the recommended guidelines for valvular heart disease.^16^^,^^17^

Surgeries were performed in the HC-UFMG Surgery Center and the valves were sent to the HC-UFMG Pathology Department for conventional histopathological examination, according to the routine established by the Cardiovascular and Cardiovascular Surgery Service of HC-UFMG, as well as the Laboratory of Molecular Pathology (LMP) of the Department of Pathological Anatomy and Legal Medicine of FM-UFMG, to carry out this study.

### Echocardiographic evaluation

Two-dimensional (2D) echocardiography imaging and Doppler was performed using a commercially available system (Philips ie33, Andover, MA or GE Vivid-q Horten, Norway) in all patients. Standard echocardiograms were obtained according to the American Society of Echocardiography guidelines.^18^ The conventional indexes for assessment of mitral stenosis severity, including mitral valve area, transmitral valve pressure gradients and pulmonary artery systolic pressure were measured, as recommended.

### Histological analysis

The valves received in the LMP were examined and referred for histological processing according to routine laboratory protocols, including fixation, paraffin embedding, hematoxylin-eosin staining and microscopic analysis. Semi-quantitative analysis of the samples was carried out using a grade scale.

Parameters of inflammation intensity, identification of the predominant cell type and fibrosis intensity were evaluated, in addition to the presence of neoangiogenesis, calcification and adipose metaplasia.

Endocardial thickness was determined in millimeters. Intensity of inflammation and fibrosis was semiquantitatively graded as absent, mild, moderate and severe by 2 independent observers with subsequent analysis by an experienced pathologist who made the final classification. The presence of neoangiogenesis, calcification and adipose metaplasia was defined as absent or present.

### Statistical analysis

Categorical variables, expressed as numbers and percentages, were compared using chi-squared testing. Normality of continuous variables was tested using the Shapiro-Wilk test, and data with normal distribution were expressed as mean±standard deviation (SD) and differences between inflammation intensity categories, valve area and leaflet thickness were assessed using Student’s unpaired t-test. Mean valve area and leaflet thickness were compared in 3 categories of inflammation, using one-way ANOVA test and post hoc analysis with Tuckey’s test. Differences that returned P values of <0.05 were considered statistically significant. Statistical analysis was performed using the Statistical Package for Social Sciences for Windows, version 22.0 (SPSS Inc., Chicago, Illinois).

## Results

### Patient characteristics

The mean age of the patients was 53±13 years and 36 (90%) were females. The characteristics of the study population comparing patients with controls are summarized in Table 1. The majority of the patients were in NYHA functional class III and IV (60%). Figure 1 shows representative pictures of the anatomical characteristics of mitral valves from controls and patients.

**Table 1:** Demographic, clinical and echocardiographic data of the patients who underwent mitral valve replacement due to rheumatic heart disease comparing with controls who underwent heart transplant

Variables*	Rheumatic mitral valves (n=40)	Controls (n=20)	p value
Age (years)	53±13.2	50±11.6	0.334
Female gender	36 (90)	6 (30)	<0.001
Functional class III/IV	24 (60)	16 (80)	0.003
Right-sided heart failure	12 (30)	12 (60)	0.030
Previous mitral valvuloplasty	22 (55)	…	…
Previous history of acute rheumatic fever	27 (68)	…	…
Use of antibiotic prophylaxis	13 (33)	…	…
Atrial fibrillation	24 (60)	5 (25)	0.003
Previous stroke	12 (30)	4 (20)	0.102
Previous hospitalization for heart failure	17 (43)	20 (100)	<0.001
**Echocardiographic parameters**			
Left ventricular end-diastolic diameter (mm)	51.7±10.9	66.3±7.6	<0.001
Left ventricular end-systolic diameter (mm)	35.8±8.6	57.1±8.2	<0.001
Left ventricular ejection fraction (%)	60.6±10.5	26.5±7.5	<0.001
Mitral valve area (cm^2^)	1.18±0.37	…	…
Left atrial diameter (mm)	55.1±10.5	48.9±6.4	0.006
Pulmonary artery systolic pressure (mmHg)	46.5±18.3	39.7±16.8	0.292
Right ventricular dysfunction	16 (40)	9 (45)	0.785

* Data are expressed as mean±SD or number (percentage) of patients.

**Figure 1:** Gross morphological aspects of the mitral valves from controls and patients with rheumatic heart disease. Note that the in rheumatic valves, and the anterior and posterior mitral leaflets are fused at the commissures. The chordae are shortened and fused.

Preoperative medications most frequently used were diuretics (60%) and beta-blockers (55%). Patients with atrial fibrillation and/or previous stroke were taking anticoagulants. Thirteen patients (33%) were using penicillin benzathine for secondary prevention of rheumatic fever. The mean age of these patients was 47.9±11.3. None of the patients included in the study had clinical evidence of active acute rheumatic fever.

Regarding the pattern of valve involvement, 18 patients (45%) had pure mitral stenosis, 14 patients (35%) had mixed mitral valve disease, and 8 patients (20%) displayed predominantly mitral regurgitation. Aortic lesion was detected in the echocardiogram of 15 patients (38%). Valvuloplasty had previously been performed in 22 patients (55%), including either percutaneous or surgical intervention. Surgical indication for complications of percutaneous valvuloplasty occurred in 6 patients (15%), 4 of whom due to severe mitral regurgitation related to leaflet or subvalvar apparatus damage. Among those who developed severe mitral regurgitation, 2 patients presented tear of the anterior leaflet and underwent emergency surgery for valve replacement. Two patients had laceration of P3 and A3 scallops in contiguity with posteromedial commissure, and posterior leaflet at central scallop location (P3), respectively, and underwent elective surgery for valve replacement. The other 2 patients presented worsening of mitral regurgitation after the procedure with suboptimal valve opening.

The control group consisted of 20 patients who underwent cardiac transplant, with mean age of 50±12 years, similar to the cases of RHD, with the majority of males (70%). The causes of heart failure were Chagas dilated cardiomyopathy (9 patients), cardiomyopathy after myocarditis (3), ischemic cardiomyopathy (3), idiopathic dilated cardiomyopathy (4) and retransplantation by autoimmune rejection to the graft (1). Secondary moderate to severe mitral regurgitation was found in 7 patients (35%). The other echocardiographic data are shown in Table 1.

### Histopathology analysis of rheumatic mitral valves

The endocardium with rheumatic mitral valves was thicker (1.3±0.5 vs. 0.9±0.4 mm) with greater intensity of fibrosis and inflammatory infiltrate compared with controls (Figures 2A and B). The histological findings of rheumatic mitral valves comparing to controls are shown in Table 2. Overall, rheumatic valves presented inflammation of mild intensity, focal distribution and predominance of mononuclear cells, and fibrosis of moderate to severe intensity (Figures 2C and D). Among patients using benzathine penicillin, 9 (69%) had mild inflammation. Neoangiogenesis was more frequent in rheumatic mitral valves than in controls (Figures 2E and F).

**Figure 2:** Representative histological image of mitral valve from controls and rheumatic heart disease patients stained with hematoxylin-eosin. A) Histological view of a control mitral valve, showing mild fibrosis (arrow). B) Rheumatic mitral valve with severe endocardial (asterisk) and interstitial (arrowhead) fibrosis. Panels C-I show specific aspects of mitral valves from rheumatic heart disease patients. C) Presence of endocardium (arrow) and valvar interstitium (asterisk). In the endocardium, there is mild fibrosis. D) Moderate endocardial fibrosis (arrowhead). Staining: hematoxylin-eosin. E) Neovascularization (arrow) with some inflammatory elements (arrowhead). F) Inflammatory foci (arrow) are detectable within neovascularization foci (asterisk). G) Nodular calcification (asterisk). H) Adipose metaplasia (arrow) with areas of fibrosis (arrowhead).

**Table 2:** Histological data of rheumatic mitral valves and controls

Variable*		Rheumatic mitral valves (n=40)	Controls (n=20)	p value
**Endocardium**				
Thickness (mm)		1.3±0.5	0.9±0.4	0.003
Intensity of inflammation	Absent or mild	9 (22)	13 (64)	0.004
	Moderate or severe	31 (78)	7 (36)	
Pattern of inflammation	Focal	31 (78)	7 (36)	0.004
Intensity of fibrosis	Absent or mild	17 (43)	19 (95)	0.001
	Moderate or severe	23 (57)	1 (5)	
Pattern of fibrosis	Even	23 (57)	0	< 0.001
	Uneven	17 (43)	7 (35)	
Neoangiogenesis		11 (28)	0	0.030
**Interstitium**				
Intensity of inflammation	Absent or mild	33 (82)	14 (72)	0.321
	Moderate or severe	7 (18)	6 (28)	
Pattern of inflammation	Focal	37 (92)	7 (36)	<0.001
Intensity of fibrosis	Absent or mild	0	14 (71)	<0.001
	Moderate or severe	40 (100)	6 (29)	
Pattern of fibrosis	Even	4 (10)	0	0.200
	Uneven	36 (90)	20 (100)	
Neoangiogenesis		24 (60)	7 (35)	0.130
Calcification		14 (35)	0	0.010
Adipose metaplasia		4 (11)	4 (21)	0.320

* Data are expressed as mean±SD or number (percentage) of patients.

None of the valves of the control group presented calcification whereas 35% of rheumatic valves had calcification (Figure 2G). Only a small portion of the valves in both groups had adipose metaplasia (Figure 2H).

According to the type of rheumatic mitral valve lesion, calcification was more frequent in pure mitral stenosis compared with mitral regurgitation or mixed lesions. Histological data according to the predominant mitral valve lesion that required valve replacement are shown in Table 3.

**Table 3:** Histological findings according to the predominant mitral valve lesion

Variable*		Pure stenosis (n=18)	Regurgitation and combined lesions (n=22)	p value
Inflammation	Absent or mild	15 (83)	18 (82)	0.900
	Moderate or severe	3 (17)	4 (18)	
Neoangiogenesis		11 (61)	13 (59)	0.897
Calcification		10 (56)	4 (18)	0.014
Adipose metaplasia		1 (6)	4 (18)	0.230

* Data are expressed as number (percentage) of patients.

We further stratified the patients into 3 groups according to inflammation intensity in low, moderate and high to correlate with the mitral valve area. We observed that the patients with a higher degree of inflammation and without calcification had a larger mitral valve area (Figures 3 A and B, respectively). There were no differences regarding leaflet thickness and inflammation intensity (Figure 3C) nor neoangiogenesis and mitral valve area (Figure 3D).

**Figure 3:** Associations between histological findings and mitral valve area and leaflet thickness. A) Mitral valve with high and moderate degree of inflammation show higher valve area compared with valves with low degree of inflammation. B) Calcification was associated with mitral valve area, indicating mitral stenosis as a predominant lesion in more calcified valves. C) Association between leaflet thickness and intensity of inflammation. D) Association between neoangiogenesis and mitral valve area.

### Echocardiographic parameters associated with histological findings

Echocardiographic parameters and histological findings were further compared. We observed an association between left ventricular systolic function, assessed by ejection fraction, and intensity of inflammation. Patients with left ventricular dysfunction, defined by ejection fraction less than 50%, showed a predominance of moderate to marked inflammation compared to patients with preserved ventricular systolic function (50% and 12% respectively, p=0.023). While valvular calcification was not associated with left ventricular dysfunction, patients with right ventricular dysfunction had a higher calcification compared with patients with normal right ventricular function (56% and 21% respectively, p=0.021), which may be related to the type of lesion associated with calcification.

## Discussion

Leaflet tissue inflammation and fibrosis play a central role to induce progressive valve damage in RHD.^13^ The present study evaluated rheumatic mitral valves in the end-stage of RHD when life-threatening valve dysfunction required surgery for valve replacement. The histological findings from rheumatic valves were compared with excised mitral valves from patients who underwent cardiac transplant, without primary valve disease.

Analysis of histological data showed marked fibrosis among all rheumatic valves, showing that continued fibrosis perpetuates throughout RHD progression. The fibrotic state of the mitral valve precedes myocardial dysfunction and manifest heart disease, making it important to develop diagnostic and therapeutic strategies to reduce structural remodeling of heart valves.^19^

Notably, even though patients are later in their disease, the valves continue to show active inflammatory processes, predominantly composed of mononuclear cells. The intensity of inflammation was associated with mitral valve area, but not with leaflet thickness. Low inflammatory intensity was found in a smaller valve area, probably due to fibrosis intensity that has an inverse association with the degree of inflammation.

Patients with left ventricular dysfunction presented a higher degree of valve inflammation, which may indicate adjacent myocarditis. However, 4 of the patients with left ventricular dysfunction had mitral insufficiency as the predominant lesion that may be the cause of the left ventricular dysfunction, which is an indication for valve intervention.^16^^,^^17^

Studies indicate that the autoimmune process involved in RHD begins when the reactive antibodies bind to the valvar endothelium, leading to inflammation and cellular infiltration. Once activated, the valvar endothelium increases the expression of adhesion molecules, which facilitates T-cell binding and infiltration.^20^ After the initial valvar insult, the process triggers a cascade leading to the recognition of additional epitopes, leading to progressive valve damage.^21^ Evidence that continued presentation of autoantigens at the lesion site contributes to an amplification of the immune response is reinforced by the significant reduction in autoantibody levels after surgical removal of the affected leaflets.^22^

After activation of the valvar endothelium with the adhesion of activated T cells, the scarring, neovascularization and lymphocyte infiltration cycle begins.^23^ Avascular valvar tissue is normally protected by the endothelium until a triggering factor, which may be antibodies and/or inflammatory cytokines, breaks the endothelial barrier, allowing the cycle of cell infiltration and healing to begin.^24^ Once initiated, the healing process becomes more intense in the valve interstitium due to the activation and proliferation of myofibroblasts responsible for valve fibrosis.^25^ It has already been demonstrated that, in view of some pathological conditions such as RHD, interstitial cells can transform into an activated myofibroblast phenotype, expressing inflammatory proteins and cytokines, capable of rapidly remodeling the extracellular environment.^26^ The significant decrease in plasma levels of biomarkers of collagen metabolism following mitral valve replacement strongly suggests the contribution of the mitral valve apparatus to the perpetuation of the fibrotic process in RHD.^19^

Calcification was detected in 35% of the rheumatic valves, predominantly in pure mitral stenosis, and correlated with valve area. Calcification was also more frequent in patients with right ventricular dysfunction. The identification of calcification reinforces the chronicity of the process, and its occurrence may be related to the mechanisms underlying the end-stage of rheumatic impairment.^27^ The predominance of calcification observed in rheumatic valves with pure stenosis and in patients with right ventricular involvement confirms that this lesion predominates in the late stages of the disease.^3^ Rajamannan et al. demonstrated that calcification occurs in areas of neoangiogenesis, stimulated by an active inflammatory process, formed especially by macrophages and myofibroblasts.^28^ Banerjee et al. found a greater degree of fibrosis and neovascularization with focal perivascular mild infiltration predominantly of lymphocytes and plasma cells.^19^

### Study limitations

The small number of patients included in the study is a limitation. However, considering that most rheumatic patients with indication for valve intervention undergo percutaneous valvuloplasty, the number included represents the totality of samples available during the study period.

The high prevalence of atrial fibrillation, previous history of stroke, previous valve intervention, limiting dyspnea (NYHA III and IV) and pulmonary hypertension have been shown to be severe in patients with advanced disease. As we included only patients with indication for valve replacement, our population is, therefore, representative of a spectrum of more severe stages of disease.

Mitral valve replacement is indicated for symptomatic patients with advanced disease and marked valve anatomy deformity, where mitral valve repair is unlikely.^29^ Therefore, the sample collected for our study represents the advanced rheumatic process, limiting the evaluation of the process in its initial phase.

The valves from the control group are not healthy, normal valves, since they were collected from patients that had undergone heart transplantation due to severe left ventricular dysfunction of different etiologies. Chagas heart disease, the predominant etiology of valves collected from transplanted hearts, is an inflammatory disease, associated with fibrosis. Although Chagas disease primarily affects the ventricular myocardium, histological valve abnormality may be associated with heart failure.^30^ In addition, 35% of patients presented moderate to severe secondary mitral valve regurgitation, which may explain abnormal histological findings. The differences in histological analysis could be even greater if compared to healthy tissue valves.

### Clinical implications

The pathological process involved in RHD is complex and still not fully understood. The identification of an active chronic inflammatory process, although of mild intensity, probably responsible for the maintenance of immune response and progression of valve lesion, provides subsidy for additional research, aiming to define strategies that can interrupt the progression of valvular damage and its consequences.

## Conclusions

Our findings demonstrate that, despite intense degree of fibrosis, the inflammatory process remains active in the rheumatic mitral valves, even at late disease process with valve dysfunction. This inflammation was associated with mitral valve area and left ventricular function. Valvular calcification was more frequent in mitral stenosis and among patients with right ventricular dysfunction, indicating late and severe rheumatic involvement. It is necessary to better understand what maintains the inflammation and how its persistence may predispose to clinical complications as well as the mechanisms that influence RHD mortality.
